# Larvicidal Activity of Synthesized Silver Nanoparticles from *Curcuma zedoaria* Essential Oil against *Culex quinquefasciatus*

**DOI:** 10.3390/insects10010027

**Published:** 2019-01-11

**Authors:** Nataya Sutthanont, Siriluck Attrapadung, Surang Nuchprayoon

**Affiliations:** 1 Interdisciplinary Program of Biomedical Sciences, Graduate School, Chulalongkorn University, Bangkok 10330, Thailand; nanzzy0704@gmail.com; 2 Lymphatic Filariasis and Tropical Medicine Research Unit, Chulalongkorn Medical Research Center, Faculty of Medicine, Chulalongkorn University, Bangkok 10330, Thailand; 3 Department of Medical Entomology, Faculty of Tropical Medicine, Mahidol University, Bangkok 10400, Thailand; at_siriluck@yahoo.com; 4 Department of Parasitology, Faculty of Medicine, Chulalongkorn University, Bangkok 10330, Thailand

**Keywords:** mosquito control, silver nanoparticles, plant-based insecticide, zedoary, white turmeric

## Abstract

*Culex quinquefasciatus* is the major vector of the bancroftian filarial parasite which causes human lymphatic filariasis and St. Louis encephalitis. The simple way to stop the transmission is to control the vector by using synthetic chemicals. However, herbal essential oils have biological properties, such as a larvicidal effect and are ecofriendly to use. In this study, we investigated the larvicidal activity of *Curcuma zedoaria* essential oil (ZEO) and biosynthesized silver nanoparticles using this essential oil (ZEO-AgNPs). The larvicidal activity against both insecticide-susceptible and -resistant strains of *Cx. quinquefasciatus* larvae of ZEO were investigated and compared with ZEO-AgNPs. The ZEO-AgNPs showed the utmost toxicity against both strains of *Cx. quinquefasciatus*. After 24 h of exposure, LC_50_ and LC_99_ of ZEO against susceptible strain were 36.32 and 85.11 ppm, respectively. While LC_50_ and LC_99_ of ZEO against the resistant strain were 37.29 and 76.79 ppm, respectively. Whereas ZEO-AgNPs offered complete larval mortality within 24 h of exposure, LC_50_ and LC_99_ of ZEO-AgNPs against the susceptible strain, were 0.57 and 8.54 ppm, respectively. For the resistant strain, LC_50_ and LC_99_ values were 0.64 and 8.88 ppm, respectively. The potency in killing *Cx. quinquefasciatus* and stability of ZEO-AgNPs have made this product a good candidate for the development of novel natural larvicides.

## 1. Introduction

Mosquitoes are serious vectors of important human parasites and microbes. These pathogens cause several diseases including malaria, encephalitis, filariasis, dengue and yellow fever, which are leading causes of death worldwide [1]. *Culex quinquefasciatus* is a domestic mosquito that exists relative to human habitation and activity. *Cx. quinquefasciatus* can spread numerous pathogens to humans and animals, including West Nile virus, St. Louis encephalitis, and lymphatic filariasis [2]. *Cx. quinquefasciatus* can also carry pathogens to livestock, birds, domestic and wild animal species such as avian malaria and zoonotic dirofilariasis that causes a loss of productivity and death [3]. Furthermore, *Cx. quinquefasciatus* is a significant nuisance, as their bites may induce local dermatitis or acute systemic allergic reactions in many people [4]. To eliminate the chain of transmission of vector-borne diseases, synthetic insecticidal chemicals have been used to control the vector for several decades [5]. However, the repeated use of synthetic agents results in lower effectiveness and ecological problems such resistance in mosquitoes, ecological unbalance and fallout to mammals [6,7]. Thus, the use of chemical vector control must be balanced against hazards towards humans, animals, non-target species, and the development of resistance [8]. The restrictions of insecticidal agents and rapid development of vector resistance which is associated with synthetic insecticides have prompted the necessity of searching for new insecticidal substances and alternative methods, as novel biological tools [9,10].

As the problem of insecticide-resistant mosquitoes to chemical agents are on the rise, natural sources, such as plant sources, are good alternatives to control mosquito vectors. They are harmless to humans and the environment, are target-specific, biodegradable and ecofriendly [11]. Natural insecticides may contain molecules with mosquitocidal effects. They have different mechanisms of action which reduce the chance of developing resistance in mosquito populations [12]. Recently, nanoparticles have proved highly effective in controlling insects. Plant-mediated synthesis of silver nanoparticles has been examined in a wide scope of entomological research because they are safe, low-cost, easily available and have a simple biosynthesis process [13,14]. Plant essential oils contain volatile compounds from different parts of plants [15], and were shown to be effective as synthetic insecticide [16,17].

*Curcuma zedoaria* (zedoary) is a member of Zingiberaceae Family. This plant originated and is widely cultivated in India, China and Southeast Asia. *C. zedoaria* is used for colds in India and to aid digestion in Indonesia and Thailand. In addition, it is used as an anticarcinogenic, antiseptic, antiperiodic, carminative agent and to support wound healing [18]. There are many reports on efficacy of *C. zedoaria* rhizome essential oil as natural larvicides against mosquito species, adulticides against both laboratory and natural field strains of *Ae. aegypti* adult females [19], as well as larvicidal and pupacidal activities against *Ae. aegypti* [20].

In this study, the larvicidal activity of *C. zedoaria* essential oil (ZEO) and silver nanoparticles synthesized with the essential oil (ZEO-AgNPs) have been investigated for toxicity against insecticide-resistant and -susceptible strains of *Cx. quinquefasciatus* larvae. UV/Visible spectrophotometry, Zetasizer, Scanning Electron Microscopy (SEM), Energy dispersive X-ray spectrometry (EDX), X-ray diffraction (XRD) and Fourier transform infrared (FTIR) spectrometry were used to confirm the expeditious biosynthesis of AgNPs.

## 2. Materials and Methods

### 2.1. Essential Oils and Chemicals

The *C. zedoaria* rhizomes were obtained from a reliable medicinal herb supplier in Bangkok, Thailand. The dried and finely ground materials of *C. zedoaria* rhizomes were extracted by hydro-distillation for the essential oil and stored at 4 °C for further experiments. The silver nitrate (AgNO_3_) was obtained from Sigma Aldrich (St. Louis, MO, USA; CAS Number: 7761-88-8). All other reagents and chemicals used were of analytical grade and bought from local agencies.

### 2.2. Mosquito Test Population and Rearing

The free-mating laboratory mosquito populations used in this research were *Cx. quinquefasciatus* deltamethrin-susceptible and -resistant strains (resistance ratio = 5.3). We obtained the mosquito colonies from the Department of Medical Entomology, Faculty of Tropical Medicine, Mahidol University, Bangkok, Thailand. The mosquitoes were reared separately in a climate-controlled insectarium (25 ± 2 °C, 70 ± 10% relative humidity, and 14:10 h, light/dark cycle) without exposure to any insecticides or pathogens. The larvae were reared in dechlorinated tap water and were fed daily on finely ground fish food.

### 2.3. Synthesis of Silver Nanoparticles

**Figure 1:**
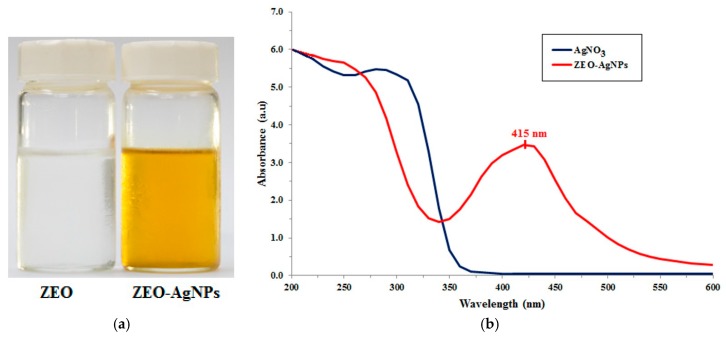
Preparation procedure of AgNPs, and UV/VIS spectroscopy. (**a**) The reactant before and then the product after the reaction. (**b**) UV/VIS spectroscopy of AgNPs. The maximum absorption peak of ZEO-AgNPs was 415 nm.

The silver nanoparticles (AgNPs) were synthesized by the essential oil reduction method of Ga’al et al. [21] with some modification. The *C. zedoaria* essential oil (ZEO) was used as both a stabilizing and reducing agent. In the biosynthesis, the ZEO in Polysorbate 20 was dissolved with deionized water. The pH value of the solution was adjusted to 7.0 using 0.1 M sodium hydroxide solution. The solution drops were dripped slowly into boiling 5 mM AgNO_3_ solution under continuous stirring. The formation of AgNPs was indicated by color change (Figure 1a).

### 2.4. Characterization of Silver Nanoparticles

The synthesized AgNPs were identified by Ultraviolet-Visible (UV/VIS) spectroscopy (Varioskan spectral scanning multimode reader, software version 2.4.5 Research Edition, Thermo Scientific, Rockford, IL, USA) at a slit width of 1 nm in the range of 200–600 nm. To evaluate the particle size, distribution was averaged using a Zetasizer Nano Instrument (Malvern, UK) at 25 °C. The synthesized AgNPs were lyophilized, then resuspended in deionized water, centrifuged at 21,420× *g* for 20 min, three times. The resulting pellet was filtered through Millipore filter paper (0.45 μm) and lyophilized again. A filtrate-containing AgNPs was used for Scanning electron microscopy (SEM), Energy dispersive X-ray spectrometry (EDX), X-ray diffraction (XRD) and Fourier transform infrared (FTIR) spectrometry.

The UV/VIS was used to analyze molecular ionic species by their absorption of particular UV or visible wavelengths in dilute solutions. The morphology of the synthesized AgNPs was detected by a scanning electron microscope (SEM), model JSM-IT500H (JEOL, Tokyo, Japan). A thin film of the sample was prepared on the stub coating with platinum. The instrument was provided with an EDX to assure the appearance of silver metal. Further, the crystalline properties of the compound were tested using XRD analysis (D8-Discover, Bruker, Billerica, MA, Germany). The XRD pattern was evaluated using a coated film of dried powder of AgNPs on glass slides. The working statuses were at a voltage of 45 keV and a current of 20 mA with Cu-Ka radiation as an X-ray source in the range of 20–80 at a 2 h angle. The FTIR spectrometry was carried out using Perkin Elmer, Spectrum One (Waltham, MA, USA) in the diffuse reflectance mode at a resolution of 4000–400 cm^−1^ in KBr pellets.

### 2.5. Larvicidal Activity

This research was approved by the Institutional Animal Care and Use Committee (IACUC) of Chulalongkorn University Laboratory Animal Center (CULAC), Bangkok, Thailand (protocol number: 1673013). The larvicidal activity was assessed by the standard protocol described by the WHO with some modification [22]. The larvicidal activity of *C. zedoaria* essential oil (ZEO) and ZEO-AgNPs were tested against both deltamethrin-susceptible and -resistant strains of *Cx. quinquefasciatus* larvae. The ZEO was diluted in dimethyl sulfoxide (DMSO) to prepare a graded dilution of concentrations. For bioassay tests, the late 3rd instar larvae of *Cx. quinquefasciatus* were tested in 4 batches of 25 larvae, with a final total of 100 larvae for each concentration. Each batch of both strains was transferred in 99 mL of deionized water and 1 mL of the desired essential oil concentration, for a total of 5 concentrations, to assess larval mortality (0–100%) within 24 h. Control and untreated larval groups received solvent-deionized water and deionized water only, respectively. The percentage mortality was reported from the average of triplicates.

For the experimental treatment of ZEO-AgNPs, each of the 4 batches of 25 late 3rd instar larvae of both *Cx. quinquefasciatus* strains were separately transferred in 99 mL of deionized water and 1 mL of the desired dosages of synthesized AgNPs were added at a total of 5 concentrations. Mortality was assessed after 24 h of exposure as 0–100%, in triplicated experiments for each concentration. The control and untreated groups were included using AgNO_3_ solution and deionized water, respectively.

### 2.6. Statistical Analysis

Mortality data were analyzed by means of computerized probit analysis. The 50% and 99% lethal concentration (LC_50_ and LC_99_, respectively) and 95% confidence intervals were derived [23] using SPSS software package version 22.0 (SPSS Inc., Chicago, IL, USA). The stability larvicidal data were analyzed using two-way ANOVA analysis with a Bonferroni post hoc test.

## 3. Results

### 3.1. Characterization of Silver Nanoparticles

The ZEO-AgNPs were synthesized through the reduction of AgNO_3_ using *C. zedoaria* essential oil (ZEO). This was a one-step AgNO_3_ reduction process. The ZEO acted as both a reducing and stabilizing agent for the formation of AgNPs. The formation of AgNPs in the reaction mixture was basically indicated by the color change from colorless to light brown (Figure 1a). The mixture of the AgNO_3_ solution and ZEO were combined under steady stirring at high temperature. The color of the solution changed within 3–4 h. The absorption of UV/VIS spectra in the wavelength ranges 200–600 nm (Figure 1b). The maximum absorption peak of ZEO-AgNPs was found at 415 nm.

The particle size of ZEO-AgNPs was measured using a Zetasizer. Measurement parameters were as follows: At 25 °C, the measurement position was 4.65 mm, the dispersant viscosity was 0.8872 mPa·s, the dispersant refractive index was 1.330 and the material refractive index was 0.140. The ZEO-AgNPs had an average size of 92.44 d·nm (6.30–167.80 d·nm) with a polydispersion index (PdI) of 0.529.

**Figure 2:**
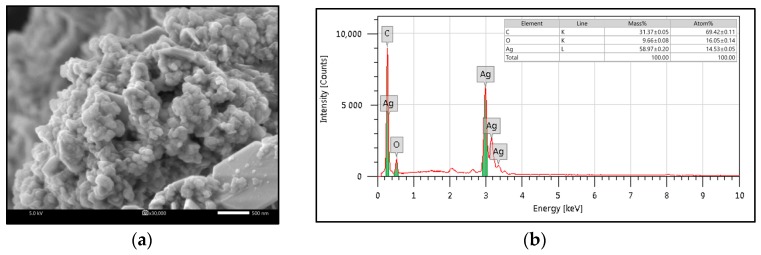
(**a**) Scanning Electron Microscopy (SEM) image and (**b**) Energy dispersive X-ray spectrometry (EDX) pattern of AgNPs synthesized with *C. zedoaria* oil (ZEO-AgNPs).

Using scanning electron microscopy (SEM, Figure 2a), ZEO-AgNPs was found to have a globular shape. As shown in Figure 2b, the element information of ZEO-AgNPs was indicated by the EDX analysis. The EDX pattern of ZEO-AgNPs was shown as a peak in the silver region. The presence of an absorption peak of the silver element was observed around 3 KeV, which demonstrates the result that silver is the main component in AgNPs (Figure 2b). These results were consistent with a successful biosynthesis of ZEO-AgNPs.

**Figure 3:**
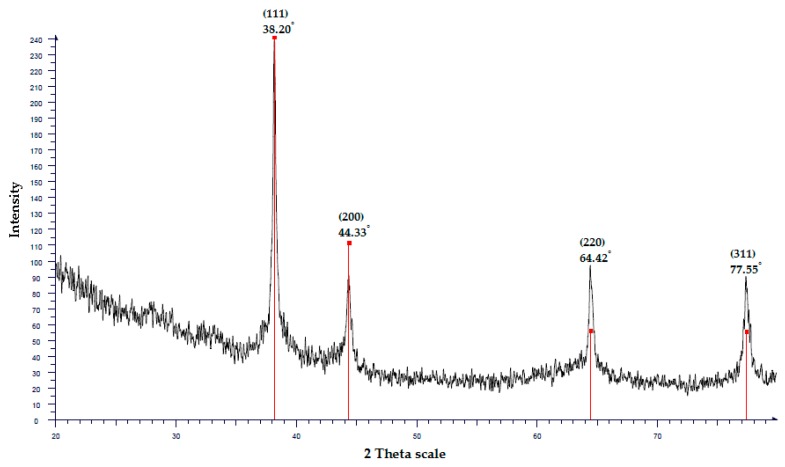
X-ray diffraction (XRD) pattern of AgNPs synthesized using *C. zedoaria* oil (ZEO-AgNPs).

To confirm the UV/VIS and EDX analysis results, X-ray diffraction (XRD) was used to verify the crystal structure of the AgNPs [24]. The XRD pattern consisted of four distinct peaks at scattering angles of 2 theta (2θ) values of 38.20°, 44.33°, 64.42°, and 77.55°, which referred to the lattice planes (111), (200), (220), and (311), respectively, which can be indexed for the cubic nature of AgNPs (Figure 3).

**Figure 4:**
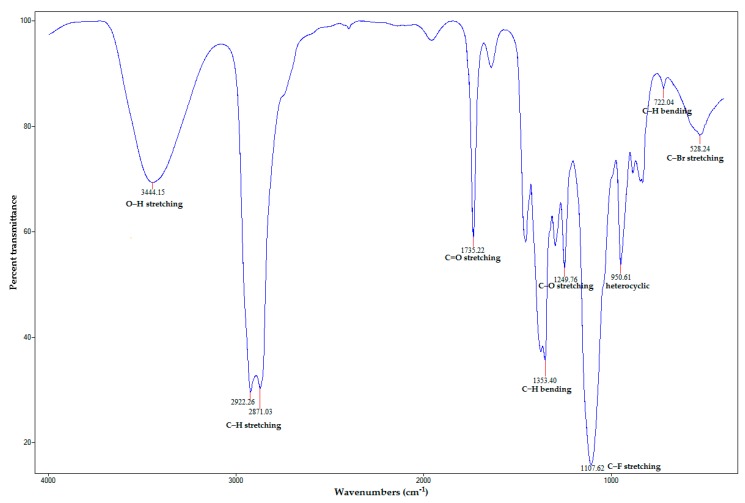
Fourier transform infrared (FTIR) absorption spectra of AgNPs synthesized using *C. zedoaria* oil (ZEO-AgNPs).

Several strong characteristic peaks of FTIR spectra are shown in Figure 4. The broad absorption peak at 3444.15 cm^−1^ was assigned to O–H stretching vibration of alcohol groups. The strong peaks at 2922.26 and 2871.03 cm^−1^ corresponded to the associated asymmetric and symmetric C–H stretching vibration of alkanes. The sharp peak at 1735.22 cm^−1^ could be assigned to C=O stretching vibration of carbonyl groups. The sharp absorption peak at 1353.40 cm^−1^ signaled to C–H bending vibration of alkanes. The peak at 1249.76 cm^−1^ represented C–O stretching vibration of acid groups. The very strong and sharp peak at 1107.62 cm^−1^ consigned to C–F stretching vibration of alkyl halides. The peak at 950.61 cm^−1^ signaled corresponding heterocyclic compounds. The band peak at 722.04 cm^−1^ exhibited the presence of aromatic C–H bending. Finally, the peak at 528.24 cm^−1^ also represented C–Br stretching vibration of alkyl halides [25,26,27,28]. Therefore, the FTIR data indicated the presence of alcohols, alkanes, aromatics, alkyl halides, carbonyl groups, and heterocyclic compounds [29]. This result indicated the several functional groups from bioactive molecules of *C. zedoaria* essential oil. Moreover, according to the study by Champakaew et al. [30] that analyzed the essential oil constituents in *C. zedoaria* rhizome from Thailand. They found β-tumerone, eucalyptol, α-zingiberene and β-sesquiphellandrene as major constituents.

### 3.2. Larvicidal Activity of ZEO and ZEO-AgNPs against Deltamethrin-Susceptible and -Resistant Strains of Cx. quinquefasciatus

The larvicidal activity of the *C. zedoaria* essential oil (ZEO) and ZEO-AgNPs were investigated under laboratory conditions. They were evaluated against the late 3rd larval stages of deltamethrin-susceptible and -resistant strains of *Cx. quinquefasciatus* (Table 1). The 24-h exposure of ZEO were satisfactorily toxic against both *Cx. quinquefasciatus* larval strains; the deltamethrin-resistant strain was slightly more resistant than the deltamethrin-susceptible strain. For the *Cx. quinquefasciatus* deltamethrin-susceptible strain, ZEO showed promising larvicidal activities with LC_50_ of 36.32 and LC_99_ of 85.11 ppm, while concentrations were 37.29 and 76.79 ppm, respectively, in the deltamethrin-resistant strain. No larval mortality was observed in the control or untreated groups.

In comparison with ZEO, the larvicidal activity ZEO-AgNPs was considerably higher against both strains of *Cx. quinquefasciatus* larvae. For the *Cx. quinquefasciatus* deltamethrin-susceptible strain, ZEO-AgNPs showed larvicidal activity at 24 h of exposure with LC_50_ of 0.57 and LC_99_ with 8.54 ppm, while concentrations were 0.64 and 8.88 ppm respectively for the deltamethrin-resistant strain. No larval mortality was observed in the control or untreated groups.

## 4. Discussion

Nanotechnology is one of the fast-growing technologies in modern science and engineering, which provides a scope for the synthesis of different particle sizes, forms, and components. The synthesis of metallic nanoparticles, such as silver, gold, and platinum have been extensively used in the control of insect vectors and pharmaceutic products [31]. In this study, a color change indicated the formation of AgNPs, when *C. zedoaria* essential oil was added to AgNO_3_. The appearance of light brown color was caused by the excitation of surface plasmon vibrations in silver nanoparticles [27] at 415 nm, which predicted the formation of AgNPs. Previous reports have shown evidence that similar color changes caused the agitation of the Surface Plasmon Resonance (SPR) peak of the synthesized AgNPs [15,21,32]. The maximum absorption peak of ZEO-AgNPs was found at 415 nm, which indicates the fabrication of AgNPs [33,34,35]. The shape of the UV/VIS peak was rather narrow, which might indicate the appearance of globular AgNPs, showing a size distribution as shown in the zetasizer and SEM image [36]. This finding is supported by similar reports in recently published studies by Ga’al et al. [21] and Basu et al. [37].

The SEM image of ZEO-AgNPs showed a globular shape, which proved the natural character of silver [21,37,38,39]. This result is consistent with the shape of the SPR peak in the UV/VIS spectra [36,40]. The noble metallic silver nanocrystals that mainly display a regular absorption peak around 3 keV, because of surface plasmon resonance [41]. The EDX pattern of ZEO-AgNPs also showed strong signs of the silver element. This 3 keV peak has been likewise published in other reports [35,42,43,44]. However, the C and O elements might come from biomolecules, such as phenolic acids, terpenoids and polysaccharides, which were tied to the exteriors of the AgNPs [36].

The XRD analysis of ZEO-AgNPs presented four major peaks (111), (200), (220), and (311), that are in the Bragg’s reflection for a given crystal lattice plane of silver nanocrystals [45]. The XRD-pattern with sharp Bragg’s peaks confirmed the crystalline cubic nature of the AgNPs [46]. This result was in conformity with many studies reporting the crystalline cubic nature of biosynthesized AgNPs [47,48,49].

The FTIR spectrum of ZEO-AgNPs can be used to analyze the possible chemical molecules that may be reliable for reducing Ag^+^ and stabilizing AgNPs in a mixed solution [21]. The presence of functional groups, such as alkanes, alkenes, sesquiterpenes, monoterpenes and carboxylic acids in the essential oil, are responsible for the reduction and stabilization of AgNPs [21,50], which might play a key role in capping and stabilization of silver ions and might be responsible for involvement in the reduction and stabilization of ZEO-AgNPs [28,51].

The mosquito larvicidal potency of ZEO (LC_50_ ranging from 36.32 to 37.29 ppm) in our study is greater than or similar to that of essential oils from the Zingiberaceae family. The volatile oil from *Zingiber officinalis* was shown to have larvicidal activity against *Cx. quinquefasciatus* with a LC_50_ value of 50.78 ppm [52]. The essential oil derived from *Alpinia speciosa*, *Cymbopogon citratus*, and *Rosmarinus officinalis* against *Ae. aegypti* larvae with LC_50_ values 0.94, 0.28 and 1.18 μL/mL, respectively [53]. The essential oils from the rhizome of *Z. nimmonii* demonstrated significant larvicidal activity against *Anopheles stephensi*, *Ae. aegypti* and *Cx. quinquefasciatus*, with LC_50_ values of 37.6 and 48.1 μg/mL, respectively. [54] In addition to *Cx. quinquefasciatus*, ZEO is also toxic to *Ae. aegypti* larvae with LC_50_ of 33.45 ppm, [30] and female adults with 5.44 μg/mg [19], *Cx. quinquefasciatus* larvae with a LT_50_ of 1.7 min [20], and *An. dirus* with a LC_50_ of 29.69 ppm [55]. Moreover, ZEO also has repellent activity against *Ae. aegypti* and *Cx. quinquefasciatus* [56]. The larvacidal activity of ZEO is likely caused by the wide variety of phytochemicals and volatile composites, such as sesquiterpenes, monoterpenes, oxygenated phenyls, carboxylic acids and carbonyl hydrocarbons [54]. The most active compound from ZEO has not been elucidated and potentially becomes a new agent for mosquito larvicides for mosquito abatement programs. Fabrication for enhancing efficacy and stability of the essential oil was then prepared by dispersing nanoparticles made from metallic silver.

The mosquito larvicidal potency of ZEO in our study is much improved in combination with AgNPs LC_50_ ranging from 0.57 to 0.64 ppm, which is an over 60-fold increase in toxicity against both strains of *Cx. quinquefasciatus* larvae. This observation is in line with other reports on the biosynthesized AgNPs showing larvicidal potentials against many mosquito vectors [35,57,58]. The silver nanoparticles synthesized from *Turbinaria ornata* were highly toxic against *Ae. aegypti*, *An. stephensi* and *Cx. quinquefasciatus* larvae with LC_50_ of 0.738, 1.134, and 1.494 μg/mL [27]. The larvae and pupae of *Ae. albopictus* were effectively killed by silver nanoparticles biosynthesized using an essential oil of *Aquilaria sinensis* (LC_50_ = 0.81–1.12 ppm) and *Pogostemon cablin* (LC_50_ = 0.85–1.19 ppm) [21]. The silver nanohybrids (AgNPs) with *Cleistanthus collinus* (Cc) or *Strychnos nuxvomica* (Sn) plant leaf extracts via microwave irradiation were highly toxic against mosquito larvae. The LC_50_ value for CcAgNPs was 11.05 mg/L to *An. stephensi* and 11.38 mg/L to *Ae. aegypti*, while SnAgNPs were 8.82 and 7.75 mg/L respectively [57]. The silver nanoparticle itself was not toxic to mosquito larvae as no mortality was seen in the control group of this study. The augmentation of toxicity of plant essential oil by AgNPs to mosquito larvae is probably due to the tiny size of AgNPs, which simply infiltrate into the exoskeleton of the insect and further into the cells of the alimentary canal, where they interfere with physiological processes [59].

## 5. Conclusions

This study is the first report on the successful synthesis of silver nanoparticles using *C. zedoaria* oil as a reducing and capping agent. Our study shows a simple, cost-effective method for the synthesis of AgNPs. The synthesized silver nanoparticles were globular in shape, soluble in water and crystalline in nature. The elemental components demonstrated the appearance of silver without any contamination. The biosynthesized silver nanoparticles augmented the larvicidal activity of *C. zedoaria* essential oil against the deltamethrin-susceptible and -resistant strains of *Cx. quinquefasciatus* larvae by 60 times. Therefore, our results show that *C. zedoaria* essential oil has the potency to be used against an insecticide-resistant mosquito vector and is a suitable candidate for the development of novel natural insecticides. Further study is needed to test the stable larvicidal potential of stored ZEO-AgNPs sample and the cytotoxic effects of ZEO-AgNPs to epithelial cells and non-target species.

## Figures and Tables

**Table 1:** Larvicidal activity of *C. zedoaria* essential oil (ZEO) and AgNPs synthesized using *C. zedoaria* oil (ZEO-AgNPs) against deltamethrin-susceptible and -resistant strains of *Cx. quinquefasciatus*.

Treatments	LC_50_ (LC_99_) ppm	95% Confidence Limit LC_50_ (LC_99_), ppm		χ^2^ (*df* = 4)	SE
		LCL	UCL		
*Cx. quinquefasciatus* deltamethrin–susceptible strain					
AgNO_3_ (control)	Nil	Nil	Nil	Nil	Nil
Deionized water (untreated)	Nil	Nil	Nil	Nil	Nil
ZEO	36.32 (85.11)	33.30 (73.28)	38.94 (107.66)	2.01	0.765
ZEO-AgNPs	0.57 (8.54)	0.49 (6.06)	0.66 (13.32)	6.37	0.137
*Cx. quinquefasciatus* deltamethrin–resistant strain					
AgNO_3_ (control)	Nil	Nil	Nil	Nil	Nil
Deionized water (untreated)	Nil	Nil	Nil	Nil	Nil
ZEO	37.29 (76.79)	35.06 (68.02)	39.405 (91.80)	2.38	0.779
ZEO-AgNPs.	0.64 (8.88)	0.55 (6.34)	0.73 (13.69)	5.93	0.139

LC_50_: Lethal concentration that kills 50% of mosquitoes; LC_99_: Lethal concentration that kills 99% of mosquitoes; LCL: lower confidence limit; UCL: upper confidence limit; χ^2^: Chi-square value; *df*: degrees of freedom; Significant at *p* < 0.05 level: SE: standard error; Nil: nil mortality.

## References

[1] Service M., Youdeowei M.S.A. (1983). Management of vectors. Pest Vector Management in Tropics.

[2] Thenmozhi V., Krishnamoorthy T., Baskarn R., Krishnamoorthi G., Balaji R., Tyagi B.K. (2014). A first note on Japanese encephalitis virus isolation from *Culex quinquefasciatus* Say in Northern West Bengal. Int. J. Mosq. Res..

[3] Subra R., Service M.W., Mosha F.W. (1984). The effect of domestic detergents on the population dynamics of the immature stages of two competitor mosquitoes, *Culex cinereus* Theobald and *Culex quinquefasciatus* Say (Diptera, Culicidae) in Kenya. Acta Trop..

[4] Peng Z., Beckett A.N., Engler R.J., Hoffman D.R., Ott N.L., Simons F.E. (2004). Immune responses to mosquito saliva in 14 individuals with acute systemic allergic reactions to mosquito bites. J. Allergy Clin. Immunol..

[5] Chareonviriyaphap T., Bangs M.J., Suwonkerd W., Kongmee M., Corbel V., Ngoen-Klan R. (2013). Review of insecticide resistance and behavioral avoidance of vectors of human diseases in Thailand. Parasites Vectors.

[6] Pavela R. (2008). Larvicidal effects of various Euro-Asiatic plants against *Culex quinquefasciatus* Say larvae (Diptera: Culicidae). Parasitol. Res..

[7] Abdul Rahuman A., Gopalakrishnan G., Venkatesan P., Geetha K. (2008). Isolation and identification of mosquito larvicidal compound from *Abutilon indicum* (Linn.) Sweet. Parasitol. Res..

[8] Gratz N.G. (1999). Space sprays for the control of *Aedes aegypti* in South-East Asia. Dengue Bull..

[9] Zaim M., Guillet P. (2002). Alternative insecticides: An urgent need. Trends Parasitol..

[10] Thomas T.G., Rao S., Lal S. (2004). Mosquito larvicidal properties of essential oil of an indigenous plant, *Ipomoea cairica* Linn.. Jpn. J. Infect. Dis..

[11] Nathan S.S., Kalaivani K. (2005). Efficacy of nucleopolyhedrovirus and azadirachtin on *Spodoptera litura* Fabricius (Lepidoptera: Noctuidae). Biol. Control.

[12] Okumu F.O., Knols B.G., Fillinger U. (2007). Larvicidal effects of a neem (*Azadirachta indica*) oil formulation on the malaria vector *Anopheles gambiae*. Malar. J..

[13] Kumar D., Kumar G., Agrawal V. (2018). Green synthesis of silver nanoparticles using *Holarrhena antidysenterica* (L.) Wall. bark extract and their larvicidal activity against dengue and filariasis vectors. Parasitol. Res..

[14] Kumar P.M., Murugan K., Madhiyazhagan P., Kovendan K., Amerasan D., Chandramohan B., Dinesh D., Suresh U., Nicoletti M., Alsalhi M.S. (2016). Biosynthesis, characterization, and acute toxicity of *Berberis tinctoria*-fabricated silver nanoparticles against the Asian tiger mosquito, *Aedes albopictus*, and the mosquito predators *Toxorhynchites splendens* and *Mesocyclops thermocyclopoides*. Parasitol. Res..

[15] Vilas V., Philip D., Mathew J. (2016). Biosynthesis of Au and Au/Ag alloy nanoparticles using *Coleus aromaticus* essential oil and evaluation of their catalytic, antibacterial and antiradical activities. J. Mol. Liquids.

[16] Ghosh A., Chowdhury N., Chandra G. (2012). Plant extracts as potential mosquito larvicides. Indian J. Med. Res..

[17] Gandhi M.R., Reegan A.D., Ganesan P., Sivasankaran K., Paulraj M.G., Balakrishna K., Ignacimuthu S., Al-Dhabi N.A. (2016). Larvicidal and pupicidal activities of alizarin isolated from roots of *Rubia cordifolia* against *Culex quinquefasciatus* Say and *Aedes aegypti* (L.) (Diptera: Culicidae). Neotrop. Entomol..

[18] Lobo R., Prabhu K.S., Shirwaikar A., Shirwaikar A. (2009). *Curcuma zedoaria* Rosc. (white turmeric): A review of its chemical, pharmacological and ethnomedicinal properties. J. Pharm. Pharmacol..

[19] Chaiyasit D., Choochote W., Rattanachanpichai E., Chaithong U., Chaiwong P., Jitpakdi A., Tippawangkosol P., Riyong D., Pitasawat B. (2006). Essential oils as potential adulticides against two populations of *Aedes aegypti*, the laboratory and natural field strains, in Chiang Mai province, northern Thailand. Parasitol. Res..

[20] Phukerd U., Soonwera M. (2013). Larvicidal and pupicidal activities of essential oils from Zingiberaceae plants against *Aedes aegypti* (Linn.) and *Culex quinquefasciatus* Say mosquitoes. Southeast Asian J. Trop. Med. Public Health.

[21] Ga’al H., Fouad H., Mao G., Tian J., Jianchu M. (2018). Larvicidal and pupicidal evaluation of silver nanoparticles synthesized using *Aquilaria sinensis* and *Pogostemon cablin* essential oils against dengue and zika viruses vector *Aedes albopictus* mosquito and its histopathological analysis. Artif. Cells Nanomed. Biotechnol..

[22] WHO (2005). Guidelines for Laboratory and Field Testing of Mosquito Larvicides.

[23] Finney D. (1971). Probit Analysis.

[24] Jyoti K., Baunthiyal M., Singh A. (2016). Characterization of silver nanoparticles synthesized using *Urtica dioica* Linn. leaves and their synergistic effects with antibiotics. J. Radiat. Res. Appl. Sci..

[25] Thakore S., Rathore P.S., Jadeja R.N., Thounaojam M., Devkar R.V. (2014). Sunflower oil mediated biomimetic synthesis and cytotoxicity of monodisperse hexagonal silver nanoparticles. Mater. Sci. Eng. C.

[26] Silverstein R.M., Bassler G.C., Bassler G.C., Morrill T.C., Morrill U.T.C. (1991). Spectrometric Identification of Organic Compounds.

[27] Deepak P., Sowmiya R., Ramkumar R., Balasubramani G., Aiswarya D., Perumal P. (2017). Structural characterization and evaluation of mosquito-larvicidal property of silver nanoparticles synthesized from the seaweed, *Turbinaria ornata* (Turner) J. Agardh 1848. Artif. Cells Nanomed. Biotechnol..

[28] Kumar K.P., Paul W., Sharma C.P. (2011). Green synthesis of gold nanoparticles with *Zingiber officinale* extract: Characterization and blood compatibility. Process Biochem..

[29] Williams T.R. (1963). Infrared absorption spectroscopy (Nakanishi, Koji). J. Chem. Educ..

[30] Champakaew D., Choochote W., Pongpaibul Y., Chaithong U., Jitpakdi A., Tuetun B., Pitasawat B. (2007). Larvicidal efficacy and biological stability of a botanical natural product, zedoary oil-impregnated sand granules, against *Aedes aegypti* (Diptera, Culicidae). Parasitol. Res..

[31] Mittal A.K., Chisti Y., Banerjee U.C. (2013). Synthesis of metallic nanoparticles using plant extracts. Biotechnol. Adv..

[32] Sheny D.S., Mathew J., Philip D. (2011). Phytosynthesis of Au, Ag and Au-Ag bimetallic nanoparticles using aqueous extract and dried leaf of *Anacardium occidentale*. Spectrochim. Acta Part A Mol. Biomol. Spectrosc..

[33] Suman T.Y., Elumalai D., Kaleena P.K., Rajasree S.R.R. (2013). GC–MS analysis of bioactive components and synthesis of silver nanoparticle using *Ammannia baccifera* aerial extract and its larvicidal activity against malaria and filariasis vectors. Ind. Crops Prod..

[34] Ramanibai R., Velayutham K. (2016). Synthesis of silver nanoparticles using 3,5-di-*t*-butyl-4-hydroxyanisole from *Cynodon dactylon* against *Aedes aegypti* and *Culex quinquefasciatus*. J. Asia-Pac. Entomol..

[35] Fouad H., Hongjie L., Hosni D., Wei J., Abbas G., Ga’al H., Jianchu M. (2018). Controlling *Aedes albopictus* and *Culex pipiens pallens* using silver nanoparticles synthesized from aqueous extract of *Cassia fistula* fruit pulp and its mode of action. Artif. Cells Nanomed. Biotechnol..

[36] Wang L., Wu Y., Xie J., Wu S., Wu Z. (2018). Characterization, antioxidant and antimicrobial activities of green synthesized silver nanoparticles from *Psidium guajava* L. leaf aqueous extracts. Mater. Sci. Eng. C Mater. Biol. Appl..

[37] Basu S., Maji P., Ganguly J. (2016). Rapid green synthesis of silver nanoparticles by aqueous extract of seeds of *Nyctanthes arbor-tristis*. Appl. Nanosci..

[38] Moldovan B., Sincari V., Perde-Schrepler M., David L. (2018). Biosynthesis of silver nanoparticles using *Ligustrum Ovalifolium* fruits and their cytotoxic effects. Nanomaterials (Basel).

[39] Shameli K., Ahmad M.B., Zamanian A., Sangpour P., Shabanzadeh P., Abdollahi Y., Zargar M. (2012). Green biosynthesis of silver nanoparticles using *Curcuma longa* tuber powder. Int. J. Nanomed..

[40] Manjamadha V.P., Muthukumar K. (2016). Ultrasound assisted green synthesis of silver nanoparticles using weed plant. Bioprocess Biosyst. Eng..

[41] Haldar K.M., Haldar B., Chandra G. (2013). Fabrication, characterization and mosquito larvicidal bioassay of silver nanoparticles synthesized from aqueous fruit extract of putranjiva, *Drypetes roxburghii* (Wall.). Parasitol. Res..

[42] Kalimuthu K., Panneerselvam C., Chou C., Tseng L.C., Murugan K., Tsai K.H., Alarfaj A.A., Higuchi A., Canale A., Hwang J.S. (2017). Control of dengue and Zika virus vector *Aedes aegypti* using the predatory copepod *Megacyclops formosanus*: Synergy with *Hedychium coronarium*-synthesized silver nanoparticles and related histological changes in targeted mosquitoes. Process Saf. Environ. Prot..

[43] Aravinthan A., Govarthanan M., Selvam K., Praburaman L., Selvankumar T., Balamurugan R., Kamala-Kannan S., Kim J.H. (2015). Sunroot mediated synthesis and characterization of silver nanoparticles and evaluation of its antibacterial and rat splenocyte cytotoxic effects. Int. J. Nanomed..

[44] Rokade A.A., Kim J.H., Lim S.R., Yoo S.I., Jin Y.E., Park S.S. (2017). A Novel Green Synthesis of Silver Nanoparticles Using *Rubus crataegifolius* Bge Fruit Extract. J. Cluster Sci..

[45] Lu H.W., Liu S.H., Wang X.L., Qian X.F., Yin J., Zhu Z.K. (2003). Silver nanocrystals by hyperbranched polyurethane-assisted photochemical reduction of Ag^+^. Mater. Chem. Phys..

[46] Bragg W.H., Bragg W.L. (1913). The reflection of X-rays by crystals. Proc. R. Soc. Lond. Ser. A.

[47] Murali Krishna I., Bhagavanth Reddy G., Veerabhadram G., Madhusudhan A. (2016). Eco-friendly green synthesis of silver nanoparticles using *Salmalia malabarica*: Synthesis, characterization, antimicrobial, and catalytic activity studies. Appl. Nanosci..

[48] Shaligram N.S., Bule M., Bhambure R., Singhal R.S., Singh S.K., Szakacs G., Pandey A. (2009). Biosynthesis of silver nanoparticles using aqueous extract from the compactin producing fungal strain. Process Biochem..

[49] Balaji D.S., Basavaraja S., Deshpande R., Mahesh D.B., Prabhakar B.K., Venkataraman A. (2009). Extracellular biosynthesis of functionalized silver nanoparticles by strains of *Cladosporium cladosporioides* fungus. Colloids Surf. B Biointerfaces.

[50] Kiran S.R., Devi P.S. (2007). Evaluation of mosquitocidal activity of essential oil and sesquiterpenes from leaves of *Chloroxylon swietenia* DC. Parasitol. Res..

[51] Rao K., Aziz S., Roome T., Razzak A., Sikandar B., Jamali K.S., Imran M., Jabri T., Shah M.R. (2018). Gum acacia stabilized silver nanoparticles based nano-cargo for enhanced anti-arthritic potentials of hesperidin in adjuvant induced arthritic rats. Artif. Cells Nanomed. Biotechnol..

[52] Pushpanathan T., Jebanesan A., Govindarajan M. (2008). The essential oil of *Zingiber officinalis* Linn (Zingiberaceae) as a mosquito larvicidal and repellent agent against the filarial vector *Culex quinquefasciatus* Say (Diptera: Culicidae). Parasitol. Res..

[53] Freitas F.P., Freitas S.P., Lemos G.C., Vieira I.J., Gravina G.A., Lemos F.J. (2010). Comparative larvicidal activity of essential oils from three medicinal plants against *Aedes aegypti* L.. Chem. Biodivers..

[54] Zhang Z., Han X.M., Wei J.H., Xue J., Yang Y., Liang L., Li X.J., Guo Q.M., Xu Y.H., Gao Z.H. (2014). Compositions and antifungal activities of essential oils from agarwood of *Aquilaria sinensis* (Lour.) Gilg induced by *Lasiodiplodia theobromae* (Pat.) Griffon. & Maubl. J. Braz. Chem. Soc..

[55] Pitasawat B., Champakaew D., Choochote W., Jitpakdi A., Chaithong U., Kanjanapothi D., Rattanachanpichai E., Tippawangkosol P., Riyong D., Tuetun B. (2007). Aromatic plant-derived essential oil: An alternative larvicide for mosquito control. Fitoterapia.

[56] Phukerd U., Soonwera M. (2014). Repellency of essential oils extracted from Thai native plants against *Aedes aegypti* (Linn.) and *Culex quinquefasciatus* (Say). Parasitol. Res..

[57] Jinu U., Rajakumaran S., Senthil-Nathan S., Geetha N., Venkatachalam P. (2018). Potential larvicidal activity of silver nanohybrids synthesized using leaf extracts of *Cleistanthus collinus* (Roxb.) Benth. ex Hook.f. and *Strychnos nux-vomica* L. nux-vomica against dengue, Chikungunya and Zika vectors. Physiol. Mol. Plant Pathol..

[58] Rajasekharreddy P., Rani P.U. (2014). Biofabrication of Ag nanoparticles using *Sterculia foetida* L. seed extract and their toxic potential against mosquito vectors and HeLa cancer cells. Mater. Sci. Eng. C Mater. Biol. Appl..

[59] Suresh U., Murugan K., Panneerselvam C., Rajaganesh R., Roni M., Aziz A.T., Naji Al-Aoh H.A., Trivedi S., Rehman H., Kumar S. (2018). *Suaeda maritima*-based herbal coils and green nanoparticles as potential biopesticides against the dengue vector *Aedes aegypti* and the tobacco cutworm *Spodoptera litura*. Physiol. Mol. Plant Pathol..

